# Molecular Characterization of a Novel Budgerigar Fledgling Disease Virus Strain From Budgerigars in China

**DOI:** 10.3389/fvets.2021.813397

**Published:** 2022-01-11

**Authors:** Xiaoliang Hu, Dongdong Cai, Siru Liu, Yan Li, Lulu Chen, Guangmei Luo, Hongli Pu, Yucan He, Xiangxiao Liu, Lili Zhao, Hongzhi Cao, Tiankuo Yang, Zhige Tian

**Affiliations:** ^1^Yibin Key Laboratory of Zoological Diversity and Ecological Conservation, Solid-State Fermentation Resource Utilization Key Laboratory of Sichuan Province, Faculty of Agriculture, Forestry and Food Engineering, Yibin University, Yibin, China; ^2^Sichuan Animal Disease Control Central, Chengdu, China; ^3^Animal Breeding and Genetics Key Laboratory of Sichuan Province, Sichuan Animal Sciences Academy, Chengdu, China; ^4^College of Veterinary Medicine, Jilin University, Changchun, China; ^5^Department of Animal Husbandry and Veterinary Medicine, Modern Agricultural College, Yibin Vocational and Technical College, Yibin, China; ^6^Aviation Medical Appraisal Center, Civil Aviation Flight University of China, Guanghan, China

**Keywords:** budgerigar fledgling disease virus, deletion, phylogenetic analysis, genotype variation, enhancer element

## Abstract

Budgerigar fledgling disease virus (BFDV) is the causative polyomavirus of budgerigar fledgling disease, an important avian immunosuppressive disease in budgerigars (*Melopsittacus undulatus*). In the current study, we explored the etiological role and molecular characteristics of BFDV. We identified a novel BFDV strain, designated as SC-YB19, belonging to a unique cluster with three other domestic strains (WF-GM01, SD18, and APV-P) and closely related to Polish isolates based on complete sequences. Sequence analysis showed that SC-YB19 had an 18-nucleotide (nt) deletion in the enhancer region, corresponding to the sequence position 164–181 nt, which differed significantly from all other BFDV strains. Based on sequence alignment, three unique nucleotide substitutions were found in VP4 (position 821), VP1 (position 2,383), and T-antigen (position 3,517) of SC-YB19, compared with SD18, WF-GM01, QDJM01, HBYM02, APV7, and BFDV1. Phylogenetic analyses based on complete sequences suggested that SC-YB19, along with the domestic WF-GM01, SD18, and APV-P strains, formed a single branch and were closely related to Polish, Japanese, and American isolates. These results demonstrate that BFDV genotype variations are co-circulating in China, thus providing important insight into BFDV evolution.

## Background

Budgerigar fledgling disease virus (BFDV), also called avian polyomavirus (APV), is the causative agent of budgerigar fledgling disease, an important immunosuppressive disease in budgerigars (*Melopsittacus undulatus*). The disease was first reported in 1981, with typical symptoms including abdominal distention, lack of down feathers on the back and abdomen, subcutaneous hemorrhage of nestlings, and acute death (1–3). Polyomaviruses have a wide host range, and have been identified in vertebrates such as humans (4, 5), bats (6, 7), non-human primates (8), and horses (9). In China, a BFDV strain, HBYM02, was first reported and isolated in Hubei Province in 1994. Since then, sporadic infections have occurred across the country, resulting in considerable losses to the budgerigar breeding industry (10).

BFDV was identified as the first non-mammalian member of the genus *polyomavirus* (11, 12). BFDV is a circular, double-stranded molecule with a 4 981-nt genome, which can be divided into early and late regions. The early region codes for two non-structural proteins, i.e., large T and small t antigens. The late region contains four structural proteins, i.e., VP1, VP2, VP3, and VP4 (13). The genome also contains four regulatory elements, i.e., promoter, polyadenylation signal, DNA replication origin, and enhancer regions (14).

In the present study, we report on BFDV infection in budgerigars from Sichuan Province, China, for the first time. To better understand the molecular characteristics of the identified strain, sequencing analysis was performed and a phylogenetic tree was constructed based on its complete genome. Our results should assist in elucidating the genetic evolution of BFDV in China.

## Methods

### Ethics Statement

No animals were sacrificed for this study.

### Clinical Cases and Virus Identification

During the spring of 2019, dozens of budgerigars died at a budgerigar breeding farm in Sichuan, China. The birds showed rapid weight loss and exhibited liver and lung congestion, splenomegaly, swollen kidneys, and liver hemorrhage. Heart, liver, lung, and fecal samples were collected from the dead budgerigars. The samples were ground and centrifuged at 8,000 rpm for 10 min. DNA/RNA was extracted from the resulting supernatants, then identified using primers (15). Polymerase chain reaction (PCR) primers were employed to amplify the complete sequence, as described previously (16). Samples were examined by blood agglutination assay, PCR, or reverse transcription (RT) -PCR for the presence of DNA or RNA viruses, including avian reovirus (17), infectious bursal disease virus (18), reticuloendotheliosis virus (19), avian influenza virus (20), Newcastle disease virus (21), avian leukosis virus (22), and avian adenovirus 4 (23), according to previously described methods.

### Sequence Alignment and Phylogenetic Analysis

Complete sequences were manually assembled using ClustalX (v1.83), Vector 10, and DNASTAR. Multiple sequence alignments were conducted using ClustalW in MEGA v6.0. Phylogenetic trees were constructed using the neighbor-joining (NJ) method in MEGA (v4.0). Bootstrap values were estimated for 1,000 replicates. The sequences obtained in this study were assembled and submitted to GenBank under accession number MT119153.

## Results and Discussion

In 2019, more than 20 two-week-old budgerigars died at a breeding farm and were thus collected for laboratory investigation. Tissue samples from the liver and lungs were only positive for BFDV. No other viruses were identified in the samples (data not shown). To analyze genomic characteristics, PCR was employed to amplify and sequence the complete viral genome (termed SC-YB19). The genome was 4,963-nt long and composed of six regions, including early, late, promoter, polyadenylation signal, DNA replication origin, and enhancer regions. The positions 80–126, 131–178, 180–193, 682–693, and 706–715 are speculated to be enhancer elements (14) (Supplementary Figure 1), which affect interactions with cellular transcription and replication factors and regulate viral and cellular gene products (24–27). We identified an 18-nt deletion (position 164–182) in the enhancer section (positions 131–178 and 180–193) in SC-YB19, but not in the other BFDV isolates (Supplementary Figures 2A,B). However, whether this deletion affects the transcription, replication, and virulence of SC-YB19 is unknown, and further experiments are required to investigate its potential influence on biological effects. In addition, compared with SD18, WF-GM01, QDJM01, HBYM02, APV7, and BFDV1, nucleotide substitutions were observed at 19 loci in VP4, VP2/VP3, VP1, non-coding region, T-antigen, and T/t-antigen of SC-YB19. Three nucleotide substitutions in VP4 (position 821), VP1 (position 2,383), and T-antigen (position 3,517) were only found in SC-YB19, not in the other domestic strains (Table 1). All nucleotide substitutions in SC-YB19 were nonsense mutations (Table 1). Based on sequence alignment, position 2488, 2572, 2677, 2758, 2920, 2959, 3256, and 4139 of SC-YB19, SD18, and WF-JM01 were identified to that of BFDV and APV-7, which were isolated from the USA and Japan, respectively, while position 623, 2488, 2572, 2677, 2758, 2920, 2959, 3256, 3739, 4139, and 4986 of QDJM01 and HBYM02 were identified to the German strains (BFDV1, BFDV4, BFDV5, PLYGEN, and NC004764) (Supplementary Figures 3, 4). These results indicate that domestic strains show genetic diversity and may have different ancestors. We suppose that BFDVs have undergone evolution in China.

**Table 1:** Point mutations in seven strains of BFDVs compared with SC-YB19.

**Nucleotide number**	**Region**	**SC-YB19**	**Nucleotide exchange compared with SC-YB19 (amino acid substitution compared with predicted amino acid sequence of SC-YB19)**					
			**SD18**	**WF-GM01**	**QDJM01**	**HBYM02**	**APV7**	**BFDV1**
386	VP4(inton)	G	G	G	G	G	G	C
387	VP4(inton)	C	C	C	C	C	C	G
623	VP4(inton)	C	C	C	T	T	T	T
821	VP4	C(123T)	T(123T)	T(123T)	T(123T)	T(123T)	T(123T)	T(123T)
1652	VP2/VP3	G(115G)	G(115G)	G(115G)	G(115G)	G(115G)	G(115G)	T(115A)
2383	VP1	T(157S)	A(157S)	A(157S)	A(157S)	A(157S)	A(157S)	A(157S)
2488	VP1	C(192G)	C(192G)	C(192G)	T(192G)	T(192G)	C(192G)	T(192G)
2572	VP1	A(220E)	A(220E)	A(220E)	G(220E)	G(220E)	A(220E)	G(220E)
2677	VP1	C(255A)	C(255A)	C(255A)	A(255A)	A(255A)	C(255A)	A(255A)
2758	VP1	A(282R)	A(282R)	A(282R)	G(282R)	G(282R)	A(282R)	G(282R)
2920	VP1	T(336D)	T(336D)	T(336D)	C(336D)	C(336D)	T(336D)	C(336D)
2959	Non-coding region	A	A	A	C	C	A	C
3256	T-antigen	G(515T)	G(515T)	G(515T)	A(515T)	A(515T)	A(515T)	A(515T)
3457	T-antigen	C(448K)	C(448K)	T(448K)	T(448K)	T(448K)	T(448K)	T(448K)
3517	T-antigen	G(428R)	T(428R)	T(428R)	T(428R)	T(428R)	T(428R)	T(428R)
3657	T-antigen	T(382K)	T(382K)	G(382Q)	G(382Q)	G(382Q)	G(382Q)	G(382Q)
3739	T-antigen	G(354T)	G(354G)	G(354G)	A(354T)	A(354T)	A(354T)	A(354T)
4139	T-antigen	G(221P)	G(221P)	G(221P)	G(221P)	A(221L)	A(221L)	A(221L)
4986	T/t-antigen	G(4L)	G(4L)	G(4L)	A(4L)	G(4L)	G(4L)	A(4L)

*Highlights indicate the mutant position*.

**Figure 1:**
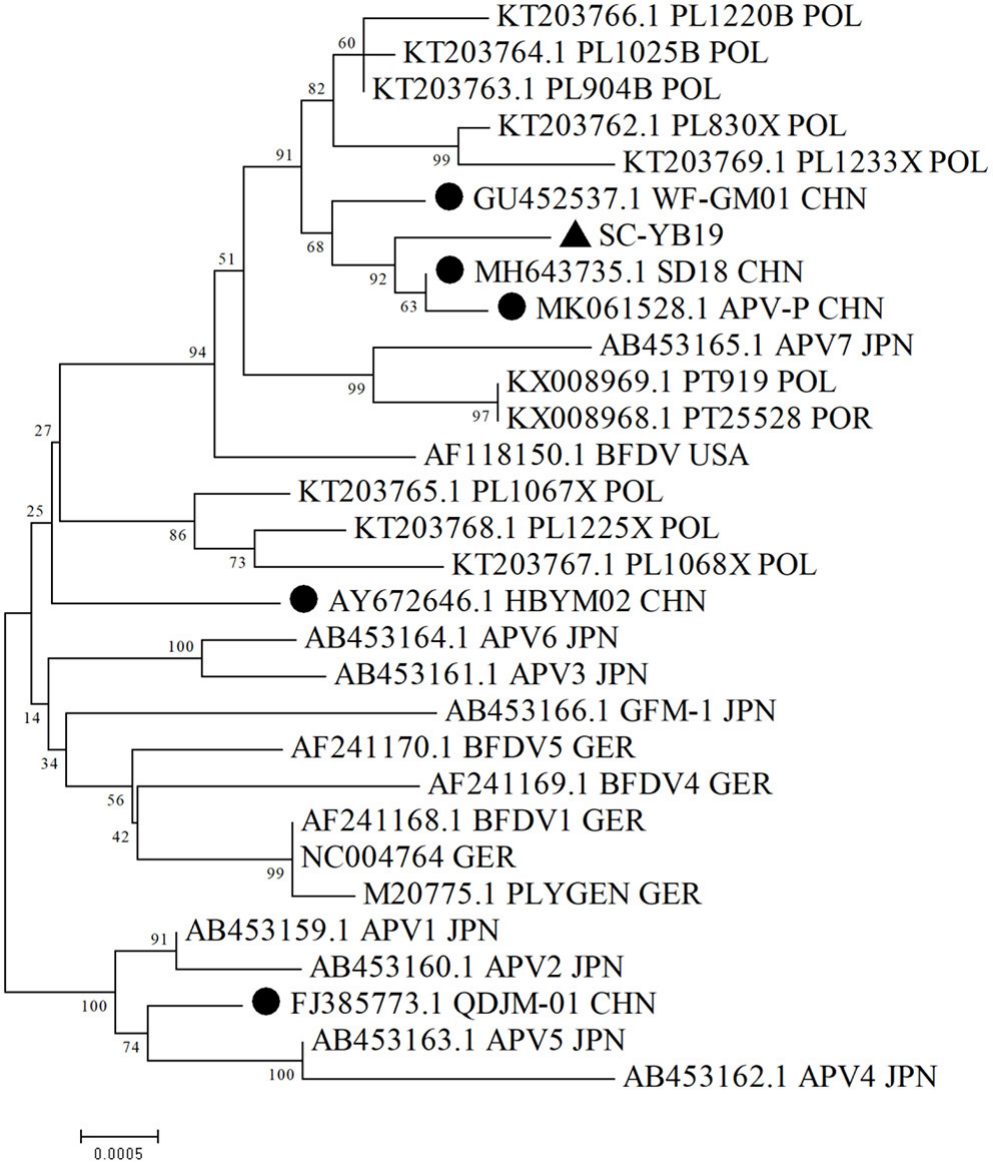
Phylogenetic analysis of complete SC-YB19 sequence and related whole-genome strains from GenBank. Neighbor-joining was used to construct a phylogenetic tree, with bootstrap values of 1,000 replicates shown on branches. Scale bar represents *p*-distance. • presents the domestic strains. ▴ presents the strains isolated in this study. CHN, China; GER, Germany; POL, Poland; POR, Portugal; USA, United States Of Amrica; JPN, Japan.

Phylogenetic analyses based on complete sequences suggested that SC-YB19, along with the domestic WF-GM01, SD18, and APV-P strains, formed a single branch and were closely related to Polish, Japanese, and American isolates (Figure 1; Table 2). QD-JM01 was clustered with the APV1, APV2, APV4, and APV5 strains isolated from the Japanese black-headed caique (*Pionites melanocephalus*). HBYM02 was distinct from the five other domestic strains and did not belong to any cluster (Figure 1). Thus, our data show that different BFDV genotypes are co-circulating in China.

**Table 2:** Source of Budgerigar fledgling disease polyomavirus (BFDV) sequences used in the experiment.

**Name**	**Genbank**	**Collection date**	**Country**
SC-YB19	MT119153	2019	CHN
BFDV1	AF241168	1984	GER
BFDV4	AF241169	1981	GER
BFDV5	AF241170	1995	GER
GFM-1	AB477106	1982	JPN
BFDV	AF118150	1999	USA
APV1	AB453159	2003	JPN
APV2	AB453160	2003	JPN
APV3	AB453161	2004	JPN
APV4	AB453162	2005	JPN
APV5	AB453163	2005	JPN
APV6	AB453164	2005	JPN
APV7	AB453165	2006	JPN
PLYGEN	M20775	1988	GER
WF-GM01	GU452537	2009	CHN
SD18	MH643735	2018	CHN
APV-P	MK061528	2018	CHN
QDJM-01	FJ385773	2008	CHN
HBYM02	AY672646	1994	CHN
PL830X	KT203762	2009	POL
PL1220B	KT203766	2010	POL
PT25528	KX008968	2015	POR
PT919	KX008969	2016	POL
PL1025B	KT203764.1	2010	POL
PL904B	KT203763.1	2009	POL
PL1233X	KT203769.1	2011	POL
PL1067X	KT203765	2010	POL
PL1225X	KT203768	2010	POL
PL1068X	KT203767	2010	POL
/	NC004764	1984	GER

## Conclusions

We report on a novel BFDV enhancer deletion mutant (SC-YB19 strain) in China. The strain forms a unique cluster with three other domestic strains. This study improves our understanding of the genetic structure, diversity, and evolution of SC-YB19.

## Data Availability Statement

The datasets presented in this study can be found in online repositories. The names of the repository/repositories and accession number(s) can be found in the article/Supplementary Material.

## Ethics Statement

The animal study was reviewed and approved by Animal Ethics Committee of Yibin University.

## Author Contributions

ZT and TY designed the study and revised the draft. XH, DC, SL, and ZT performed the experiments. YL, LC, GL, HP, YH, XL, LZ, and HC collected the samples. ZT, XH, and TY drafted the manuscript. All authors revised and approved the paper for publication.

## Funding

This work was supported by the Doctor Launch Project of Yibin University (2019QD09 and 2019QD10) and Foundation of General Program in Civil Aviation Flight University of China (J2021-134).

## Conflict of Interest

The authors declare that the research was conducted in the absence of any commercial or financial relationships that could be construed as a potential conflict of interest.

## Publisher's Note

All claims expressed in this article are solely those of the authors and do not necessarily represent those of their affiliated organizations, or those of the publisher, the editors and the reviewers. Any product that may be evaluated in this article, or claim that may be made by its manufacturer, is not guaranteed or endorsed by the publisher.

## Supplementary Material

Supplementary Figure 1 Draft enhancer element of SC-YB19 (in red box).

Supplementary Figure 2 **(A)** 18-nt deletion in the enhance element at nucleotide position 164–182 of the SC-YB19; **(B)** Result of the ABI sequencing for SC-YB19.

Supplementary Figure 3 Nucleotide sequences alignments between SC-YB19 and SD18, WF-GM01, QDJM01, AY672646, AF118150, APV-7, AF241168, AF241169, AF241170, M20775, and NC004764.

Supplementary Figure 4 Multiple sequence alignment of complete sequences in BFDV strains.

## Abbreviations

BFDV: Budgerigar fledgling disease virus
APV: avian polyomavirus
NJ: neighbor-joining
nt: nucleotides
PCR: polymerase chain reaction
RT: reverse transcription.
