# Supplementary figures and images for: Molecular Characterization of a Novel Budgerigar Fledgling Disease Virus Strain From Budgerigars in China
Source: Front Vet Sci. 2022 Jan 11;8:813397. doi: 10.3389/fvets.2021.813397 (PMC8787288; doi:10.3389/fvets.2021.813397)

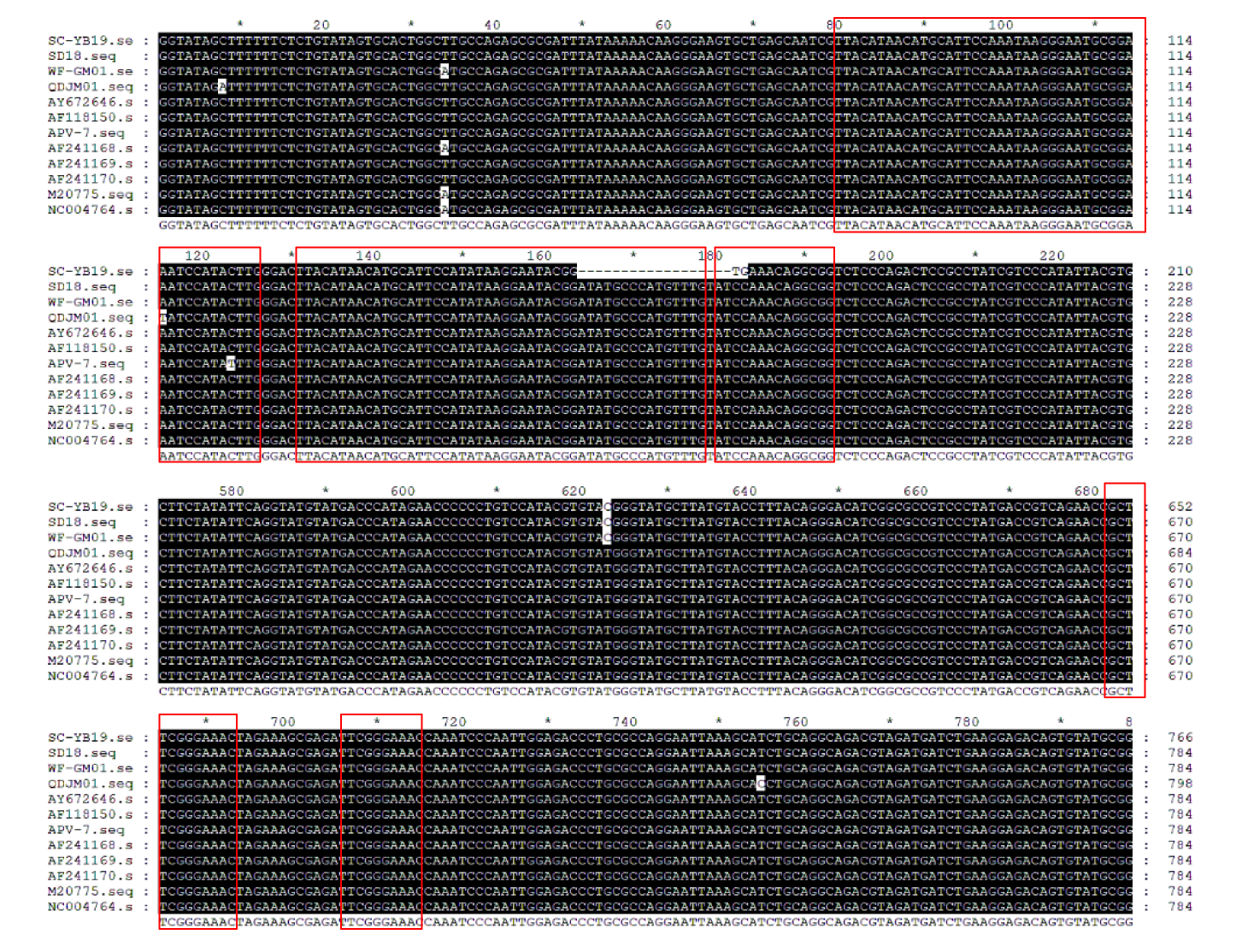

Supplement: Supplementary Figure 1 — Draft enhancer element of SC-YB19 (in red box). [file Image_1.TIF]

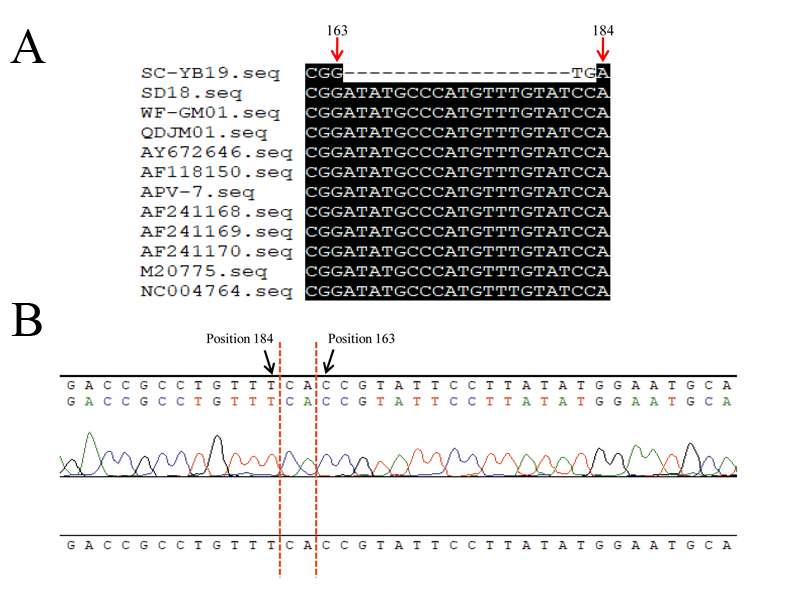

Supplement: Supplementary Figure 2 — (A) 18-nt deletion in the enhance element at nucleotide position 164–182 of the SC-YB19; (B) Result of the ABI sequencing for SC-YB19. [file Image_2.TIF]

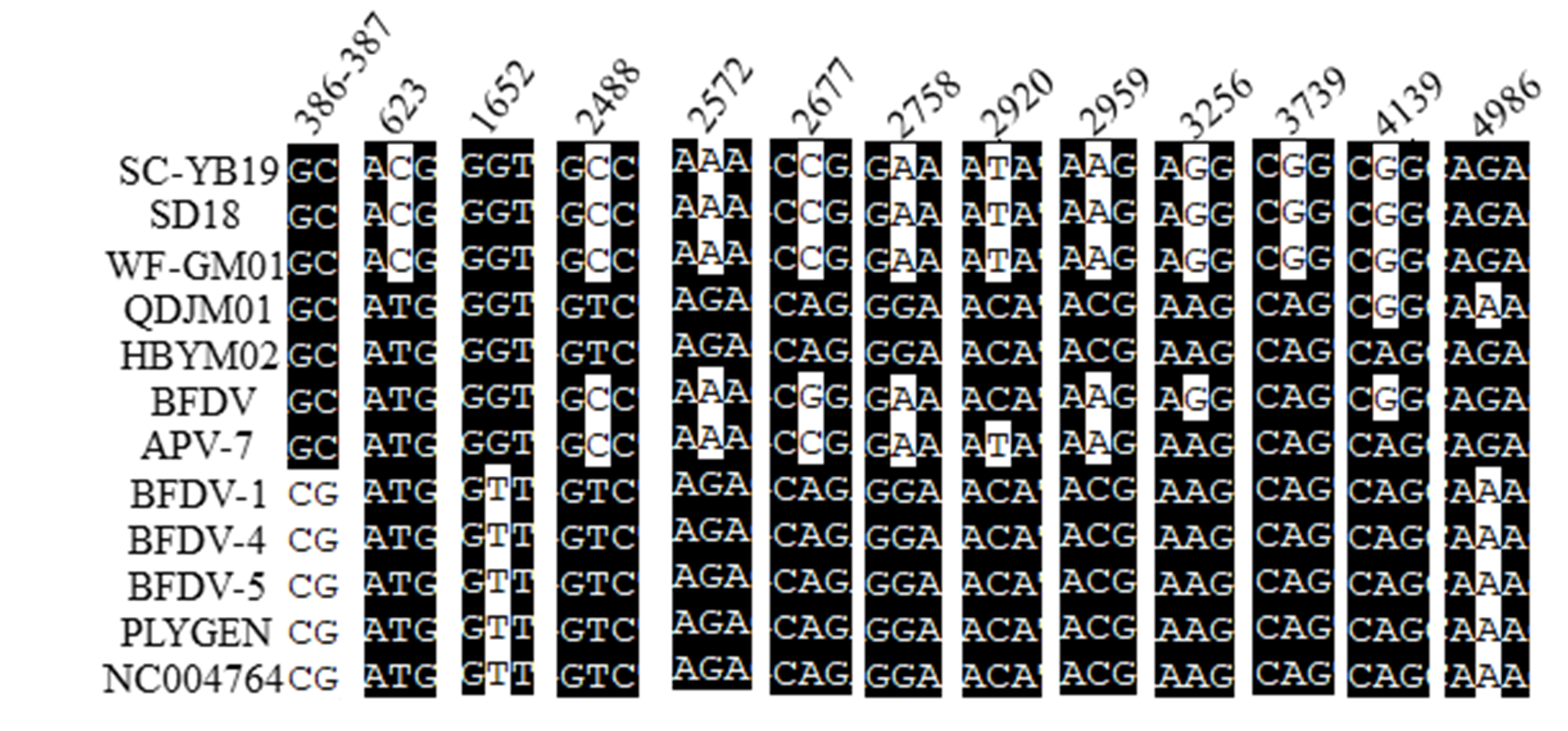

Supplement: Supplementary Figure 3 — Nucleotide sequences alignments between SC-YB19 and SD18, WF-GM01, QDJM01, AY672646, AF118150, APV-7, AF241168, AF241169, AF241170, M20775, and NC004764. [file Image_3.TIF]

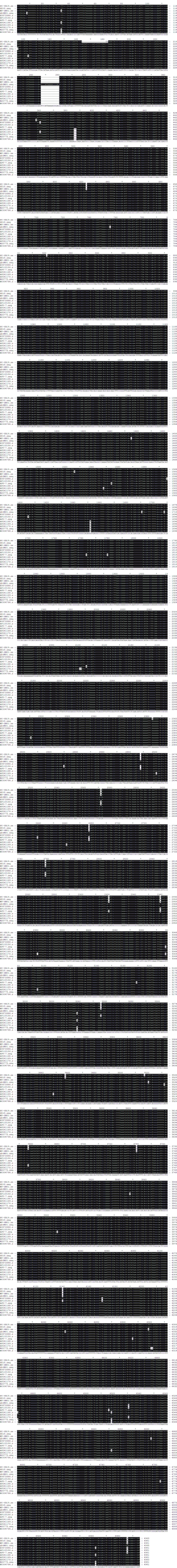

Supplement: Supplementary Figure 4 — Multiple sequence alignment of complete sequences in BFDV strains. [file Image_4.TIF]
